# Socio-Emotional Development Following Very Preterm Birth: Pathways to Psychopathology

**DOI:** 10.3389/fpsyg.2016.00080

**Published:** 2016-02-12

**Authors:** Anita Montagna, Chiara Nosarti

**Affiliations:** ^1^Department of Perinatal Imaging and Health, Centre for the Developing Brain, St. Thomas' Hospital, King's College LondonLondon, UK; ^2^Department of Psychosis Studies, Institute of Psychiatry, Psychology and Neuroscience, King's College LondonLondon, UK

**Keywords:** preterm, socio-emotional, brain, pain, stress, parenting

## Abstract

Very preterm birth (VPT; < 32 weeks of gestation) has been associated with an increased risk to develop cognitive and socio-emotional problems, as well as with increased vulnerability to psychiatric disorder, both with childhood and adult onset. Socio-emotional impairments that have been described in VPT individuals include diminished social competence and self-esteem, emotional dysregulation, shyness and timidity. However, the etiology of socio-emotional problems in VPT samples and their underlying mechanisms are far from understood. To date, research has focused on the investigation of both biological and environmental risk factors associated with socio-emotional problems, including structural and functional alterations in brain areas involved in processing emotions and social stimuli, perinatal stress and pain and parenting strategies. Considering the complex interplay of the aforementioned variables, the review attempts to elucidate the mechanisms underlying the association between very preterm birth, socio-emotional vulnerability and psychopathology. After a comprehensive overview of the socio-emotional impairments associated with VPT birth, three main models of socio-emotional development are presented and discussed. These focus on biological vulnerability, early life adversities and parenting, respectively. To conclude, a developmental framework is used to consider different pathways linking VPT birth to psychopathology, taking into account the interaction between medical, biological, and psychosocial factors.

## Introduction

Very preterm birth (VPT; < 32 weeks of gestation) is common and represents up to 13% of all births (Goldenberg et al., 2008). In the last decades, significant improvements in the management of high risk pregnancies has led to higher survival of babies born very preterm (Fanaroff et al., 2003). However, such improved survival has been associated with an increase in prevalence of neonatal problems and long-term morbidity, as very preterm infants are at greater risk of brain damage and consequent neurological disorders, neuropsychological, and behavioral impairments in childhood and later in life (Delobel-Ayoub et al., 2009; Nosarti et al., 2012; Arpi and Ferrari, 2013; Ball et al., 2013; Anderson, 2014).

Approximately 5–15% of VPT children experience motor and sensory deficits, such as cerebral palsy, vision, and hearing problems (Wood et al., 2005; Woodward et al., 2006) and more than 50% of children born extremely preterm (≤ 26 gestational weeks) experience academic difficulties (Johnson et al., 2009), with lower scores in reading, spelling, and arithmetic (Aarnoudse-Moens et al., 2009; Hutchinson et al., 2013). Together with these impairments, behavioral problems are reported in about 25% of VPT children, which can be summarized into a fairly consistent behavioral phenotype, characterized by inattention, anxiety, socio-emotional, and internalizing problems (Johnson and Marlow, 2011; Arpi and Ferrari, 2013). Such behavioral phenotype manifests in increased rates of subclinical symptomatology that, at the furthest end of the distribution, meet clinical criteria (Elgen et al., 2002; Johnson and Marlow, 2011). VPT children are thus at higher risk than controls of developing psychiatric disorders, including attention deficit and hyperactivity disorder (ADHD), autism spectrum disorder (ASD), anxiety and depression (Johnson and Marlow, 2011; Treyvaud et al., 2013) while VPT adults show a significant increase in the prevalence of ADHD (Halmøy et al., 2012), ASD (Moster et al., 2009), non-affective psychosis (Nosarti et al., 2012), depression, anxiety (Walshe et al., 2008), eating disorder (Micali et al., 2015), and bipolar affective disorder (Abel et al., 2010; Nosarti et al., 2012).

Early life deficits in socio-emotional processing have been studied as antecedents of later subclinical behavioral and psychiatric problems (e.g., Briggs-Gowan and Carter, 2008). Furthermore, research has suggested that social functioning plays a clinically relevant role in predicting the course and outcomes of several psychiatric disorders (Cannon et al., 1997; Van Os et al., 2010). Animal and human studies have put forward the hypothesis that social defeat and social exclusion, defined here collectively as the “negative experience of being excluded from the majority group” (Selten et al., 2013), as well as chronic psychosocial stress, may act as risk factors for psychiatric disorders. This hypothesis is based on the observation that individuals described as having atypical social development, poor social competence and high social anxiety are at greater risk of developing psychopathology than typically developing individuals, both in childhood and in adult life (Cannon et al., 1997; Selten and Cantor-Graae, 2005; Van Os et al., 2010; Van Os, 2013).

Atypical socio-emotional development has been described in VPT individuals (Hille et al., 2001; Bhutta et al., 2002) as early as the first year of life (Landry et al., 1990). Since socio-emotional functioning involves the ability to learn to successfully interact and communicate within a social context and to efficiently deal with emotions, it requires a skilful coordination of multiple processes (Iarocci et al., 2007; Happé and Frith, 2014), comprising social information processing, together with several other cognitive, perceptual and motor skills. It has been suggested that possible underlying causes of atypical socio-emotional development in VPT samples include deficits in basic cognitive skills: for instance, impairments in attention orienting may affect later development of gaze-following, leading to impairments in socio-communicative abilities (Karmiloff-Smith et al., 2012). Likewise, deficits in multisensory processing may affect speech perception, subsequently resulting in difficulties in communication and social interactions (Wickremasinghe et al., 2013; Stevenson et al., 2014).

In this paper, based on findings linking socio-emotional behavioral problems and psychopathology and showing high prevalence of such problems in VPT samples, we will explore possible underlying mechanisms linking VPT birth, socio-emotional vulnerability and mental health outcomes. We will focus on selective biological and environmental factors associated with VPT birth that during the course of development may interact with and affect socio-emotional processing, including structural and functional alterations in brain areas involved in processing emotions and social stimuli, perinatal stress and pain and parenting strategies.

Very preterm birth has been described as a complex amalgam of destructive and developmental disturbances, which may result in altered maturation of the highly-vulnerable developing brain (Volpe, 2009). Therefore, at least some of the behavioral and psychiatric problems described in VPT samples may be associated with differences in neurodevelopment, the life-long process by which the brain grows and adapts to change. Specific volumetric alterations have been described in brain areas involved in socio-emotional processing in VPT individuals (Peterson et al., 2000; Gimenez et al., 2006; Nosarti et al., 2008, 2014; Gousias et al., 2012; Rogers et al., 2012), although only a few studies to date have directly explored such brain alterations in conjunction with socio-emotional behavioral outcomes (e.g., Healy et al., 2013). Recent studies focusing on patterns of connectivity throughout the brain have identified alterations in thalamocortical and corticostriatal connections in VPT samples (Ball et al., 2015; Karolis et al., 2016), which are known to be crucial for social and emotional processing (Pauly et al., 2008), behavioral flexibility (Makinson and Huguenard, 2014), as well as high-order executive functions (Eisenberg and Berman, 2010). Functional alterations in these specific brain regions and connections have been described in neuropsychiatric disorders characterized by deficits in self-regulation and attention (Clerkin et al., 2013), suggesting that high order cognitive and emotional processes are subserved by dynamic interactions between brain networks, none of which is specifically “cognitive” or “affective” (Pessoa, 2008). In fact, impaired general cognition has been related to and associated with behavioral problems in VPT populations (Bayless et al., 2008; Mansson et al., 2014).

Another factor thought to affect socio-emotional development of infants born preterm is their exposure to early life stress and pain during their life-saving stay in the neonatal intensive care unit (NICU). VPT infants are exposed to repeated procedural pain-related stress during a period of rapid brain maturation and physiological vulnerability, and several studies have shown an impact of these procedures on altered neurodevelopment and hypothalamic-pituitary-adrenal (HPA) programming (Grunau, 2013; Brummelte et al., 2015), including greater internalizing behaviors in childhood (Vinall et al., 2013; Ranger et al., 2014).

A further important potential explanatory factor for impaired socio-emotional development in VPT individuals may involve the effects of early parental behavior. Life threating events in the perinatal period and the immature behavioral organization of preterm born infants may increase overall parental psychological distress and alter the interaction between child and primary carer, potentially interfering with the establishment of long-term positive parent-child relational patterns. Parental mental health has in fact been associated with children's early socio-emotional development, as it plays a pivotal role in protecting against the effects of early stress (Vinall and Grunau, 2014) and scaffolding the development of affective self-regulation (Feldman, 2007).

In this paper we will begin with a summary of socio-emotional behavioral problems in VPT infants, children and adults and describe psychiatric disorders which have been associated with VPT birth. We will consider the possible antecedents of such problems, especially impairments in social information processing, and in related cognitive and motor domains. We will then review the findings of neuroimaging studies which have investigated socio-emotional behavioral problems and psychiatric outcomes in VTP samples. We will also review some literature focusing on potential environmental influences on socio-emotional behavioral problems, such as exposure to neonatal pain and early parental behavior.

To conclude, we will consider the interplay between biological vulnerabilities, early life stress and early parental behavior in order to elucidate possible developmental mechanisms linking VPT birth, socio-emotional development and psychopathology.

## Socio-emotional behavioral problems in very preterm born individuals

The term “behavior problems” defines a wide spectrum of difficulties in behavioral self-regulation, comprising attention, and hyperactive/aggressive behaviors; sleep, eating, and sensory sensitivity problems; as well as problems with peers, anxiety, depression, and somatic symptoms. Well-validated behavioral screening questionnaires are usually administered to investigate behavioral morbidity and these tools, such as the Child Behavioral Check List (CBCL; Achenbach, 1991, 1992) and the Strengths and Difficulties Questionnaire (SDQ; Goodman, 1997), provide time- and cost-efficient measures for large-scale use (Johnson and Marlow, 2011).

Despite a relative paucity of studies in preterm-born individuals in infancy and adulthood, with most investigations focusing on school-age samples, behavioral problems have been reported as early as during the first 2 years of life (see Arpi and Ferrari, 2013 for review). However, VPT birth seems not to confer a risk for generalized behavioral morbidity, but appears to be associated with a specific triad of behavioral outcomes, characterized by inattention, emotional, and socialization difficulties, and with a greater risk of internalizing problems. The consistency of this behavioral profile in VPT samples has led to definition of a “preterm behavioral phenotype” (Johnson and Marlow, 2011).

Whilst a greater emphasis has been placed in the literature on attention problems, several studies have described socialization and emotion problems in VPT samples from infancy to adult life. The importance of a successful socio-emotional development for adult psychosocial adjustment has only recently been recognized, with studies showing a foundational contribution of socio-emotional development to later adaptive functioning, academic achievement and mental health (Emde et al., 1993; Shonkoff and Phillips, 2000). For instance, researchers have shown that more than half of typically developing children, identified as having socio-emotional problems during school-age, were already experiencing similar difficulties earlier in life, at 12–36 months (Briggs-Gowan and Carter, 2008). In the following section we will summarize the results of studies linking early socio-emotional difficulties to later well-being in VPT samples.

### Socio-emotional behavioral problems in infancy (0–2 years)

Although it is difficult to recognize behavioral problems during children's early developmental stages, due to their heterogeneous and highly changeable nature, the few studies conducted to date have shown a consistent pattern of impaired socio-emotional development following VPT birth (Arpi and Ferrari, 2013). Poorer emotional regulation and orientation/engagement was described in infants and toddlers born VPT and/or with a very low birth weight (VLBW; ≤ 1500 grams) compared to controls (Wolf et al., 2002; Janssens et al., 2009). VPT toddlers were further found to experience increased internalizing, emotion dysregulation problems and lower social competence compared to their age-matched peers (Clark et al., 2008; Spittle et al., 2009; Table 1).

**Table 1:** **Socio-emotional behavioral problems in VPT infants and toddlers**.

**Authors**	**Cases (n)**	**Controls (n)**	**Materials**	**Age (months)**	**Results**
Clark et al., 2008	39 (<28 w) 55 (<34 w)	103	ERC	24, 48	Poorer emotion regulation for lower gestational age
Janssen et al., 2008	437 (≤32 w)	0	BRS-BSID-II	29	Lower orientation/engagement in VPT group compared to the normative scores
Mansson et al., 2014	344 (<27 w)	338	CBCL	36	More internalizing problems, anxiety, depression, and social withdrawal in EPT group
Spittle et al., 2009	188 (<30 w)	70	ITSEA	24	Increased internalizing, emotion dysregulation problems, and lower social competence in VPT toddlers
Stoelhorst et al., 2003	158 (<32 w)	0	CBCL	24	Association between anxiety, depression, and social withdrawal problems and lower gestational age and increasing neurological abnormalities
Wolf et al., 2002	20 (<32 w)	10	BRS-BSID-II	3, 6	Lower emotional regulation and orientation/engagement in VLBW group

*BRS-BSID-II, Behavioral Rating Scale from Bailey Scale of Infant Development, 2nd edition; CBCL, Child Behavior Checklist; ERC, Emotion Regulation Checklist; ITSEA, Infant Toddler Socio-Emotional Assessment*.

### Socio-emotional behavioral problems in childhood

A large number of studies in preterm-born children have described a behavioral profile that seems consistent with the preterm behavioral phenotype described earlier, and we invite the reader to refer to three meta-analyses for further details (Bhutta et al., 2002; Aarnoudse-Moens et al., 2009; Arpi and Ferrari, 2013). According to published findings, very preterm-born children show significant emotional difficulties relative to peers and these difficulties are identified by elevated scores on CBCL anxiety/depression and SDQ emotional difficulties scales.

Socialization and peer relationships represent another area of concern in VPT children, which are characterized by impaired social skills and social withdrawal. During preschool- and school-age, VPT children consistently show higher scores on peer problems (SDQ) and social withdrawal (CBCL) subscales and are described as being “not liked by peers,” “rather solitary, tend to play alone,” “too dependent.” Similar findings were reported in geographically diverse samples: social problems scores, as measured by the CBCL, were higher in four population-based cohorts of extremely low birth weight children (ELBW; ≤ 1000 grams) born in 1977–1987 compared to controls (Hille et al., 2001). More recently, screening questionnaires for ASD found that VPT children compared to controls displayed significantly more symptoms reflecting social difficulties, i.e., higher rates of social and communication problems (Williamson and Jakobson, 2014a,b in children; Wong et al., 2014 in toddlers). It is also worth noticing that the majority of studies describe VPT children as having higher group mean scores on socio-emotional scales than term-born peers, even when such scores do not reach clinical cut-offs. Such findings highlight the importance of using dimensional measures of symptomatology to describe the pattern of impairments observed in VPT populations.

While published studies agree about the presence of socio-emotional difficulties in VPT samples, there seems to be less consensus on the prevalence of internalizing versus externalizing behaviors. Whereas the former is a term used to describe problems such as social withdrawal, somatic complains, anxiety, and depression, the latter refers to delinquent and aggressive behaviors (Achenbach, 1991). For further discussion on internalizing versus externalizing behaviors in VPT samples we would like to refer the reader to Johnson and Marlow (2011), Bhutta et al. (2002), Aarnoudse-Moens et al. (2009) and Arpi and Ferrari (2013).

### Socio-emotional behavioral problems in adolescence

Adolescence refers to the period of life that marks the transition from childhood to adulthood, and is a critical stage of development associated with dramatic cognitive, physical, and emotional changes, during which several brain regions underlying psychosocial and executive functions reach maturity. The biological and physical transitions associated with puberty happen in concomitance with major socio-emotional changes, such as the shift from dependency on parents to autonomy, increased educational and societal demands, changes in social affiliations and expectations. During adolescence, socialization abilities become increasingly sophisticated and assume a crucial role in facilitating social interactions including social acceptance and the attainment of social dominance.

Pre-existing socio-emotional vulnerability is then expected to become more evident during this time, and this may be why adolescence is regarded as a key developmental stage in terms of emergence and early expression of behavioral issues. It is during this period that social rejection and peer victimization become critical risk factors for psychopathology, and the increased complexity of the social network in which the adolescent finds him/herself is thought to further exacerbate pre-existing socialization problems.

Studies of behavioral problems in VPT adolescents are limited, but seem to suggest a persistence of significant behavioral difficulties, showing a 3- to 8-fold increased risk compared to term controls (using a cut-off of >90th percentile for defining clinically significant behavioral problems; Johnson and Wolke, 2013 please refer to Table 2 for a list of studies describing socio-emotional behavioral problems in adolescents). Patterns of impairment observed in preterm adolescents seem consistent with the “preterm behavioral phenotype,” despite variability in prevalence estimates for different behavioral problems. VPT adolescents have been described as being more socially isolated, as having lower self-esteem (Rickards et al., 2001) and higher social problems scores than controls (Levy-Shiff et al., 1994; Grunau et al., 2004; Indredavik et al., 2005; Hille et al., 2008; Healy et al., 2013). Furthermore, they have been found to show higher incidence of “autistic-like” traits (Indredavik et al., 2004).

**Table 2:** **Socio-emotional behavioral problems in VPT adolescents**.

**Author**	**Cases (n)**	**Controls (n)**	**Materials**	**Age (years)**	**Results**
Botting et al., 1997;	129 (≤1250 grams)	150	MFQ CMAS-R CAPA RPQ CS	12	An increased proportion of VLBW children were found to meet clinical criteria for generalized anxiety disorder. MFQ: VLBW children showed a trend toward increased depression and higher scores. RPQ: higher scores in the VLBW group.
Cooke, 2004	79 (≤1500 grams)	71	SF-36 SAS HADS Questionnaire about alcohol and drug use, sexual activity, relationships, pregnancy, involvement with the police, current height and weight, self-image, medications, academic achievements, household structure, and employment status.	19–22	VLBW and term controls showed similar quality of life and social activities. VLBW individuals drank less alcohol, used fewer illicit drugs, but smoked as often. Rates for sexual intercourse were similar. The VLBW group was shorter than controls and less satisfied with their appearance. They were more likely to use a regular prescription medicine. Fewer were or had been in higher education, and some remained unemployed.
Dahl et al., 2006	99 (≤1500 grams)	Normative population	YSR CBCL compiled by both parents and adolescents	13–18	YSF and self-rated CBCL: no significant between group differences. Parental CBCL: more social and attention problems and less social and school competence in VLBW boys and more internalizing behavior and social and attention problems and less school competence in VLBW girls. Higher likelihood for VLBW adolescents to be in the borderline/clinical range on all of the scales, except for externalizing behaviors and social problems in girls.
Farooqi et al., 2007	86 (<26 w)	86	CBCL TRF DSRS	11	Parents and teachers rated the preterm group as having more internalizing problems (anxious/depressed, withdrawn, or somatic problems) and attention, thought, and social problems. DSRS: higher scores for the preterm group, indicating a trend toward increased depression symptoms, compared with controls. No differences in the number of individuals scoring in the abnormal range.
Gardner et al., 2004	179 (<29 weeks)	108	SDQ MFQ YSR Self-esteem questions Competence scales of CBCL	15–16	Parents and teachers rated extremely preterm adolescents as scoring in the abnormal range on hyperactivity, emotional and peer problems but not on conduct problems. No differences in self-ratings of hyperactivity, peer or conduct problems. Extremely preterm adolescents were more likely to score themselves as having more emotional problems.
Grunau et al., 2004	53 (≤800 gr)	31	SPPA CBCL	17	ELBW teens reported more Internalizing, Externalizing, and Total Problems. More problems above the clinical cut-off for Total, Internalizing, and Externalizing Problems.
Healy et al., 2013	73 (<33 w)	49	CBCL CAPA CIS- R	15, 19	At 14-15, VPT individuals had higher scores on the CBCL social problems scale and were almost 4 times more likely to fall into the socially immature group compared to controls. At 19, VPT individuals had slightly higher CIS-R total scores compared with controls. Social immaturity predicted higher CIS-R, irrespective of group membership.
Hille et al., 2008	656 (<32 w)	General population/normative data	YASR YABCL National registers	19	Boys had more trouble in establishing a relationship. No difference in clinical psychopathology but VPT individuals showed higher scores compared to norms in most of the YABCL mean problem scores.
Indredavik et al., 2005	55 (≤ 1500 grams) 55 (term small for gestational age)	66	SADSSAC ASSQ ADHD-Rating Scale IV	14–15	VLBW and term small for gestational age adolescents manifested increased prevalence of psychiatric symptoms and disorders. VLBW showed more symptoms of ADHD and anxiety. Higher mean ASSQ sum score.
Levy-Shiff et al., 1994	90 (≤1500 grams and < 35 w)	90	STAIC CDI CAI TSCS CBI CSQ	13–14	VLBW individuals were emotionally less well-adjusted, more anxious, more depressed, more aggressive, and with lower self-concepts. Reported by their parents and teachers to manifest more behavioral disturbances at home and at school.
Rickards et al., 2001	130 (≤1500 grams)	42	ATRS CBCL CSEI YSR	14	ATRS: VLBW group more socially rejected. CBCL: No significant group differences. CSEI: VLBW group significant lower level in the General Self Subscale. Differences in how they feel about themselves, lower self-esteem.
Saigal et al., 2003	141 (<1000 grams)	122	OCHS-R	12–16	Higher depression subscale scores and ADHD in ELBW adolescents.
Stevenson et al., 1999	132 (≤1500 grams)	132	RPQ RTQ CSQ	8, 14	More emotions and behavioral problems in VLBW adolescents.

*ADHD-Rating Scale IV, Attention-Deficit/Hyper- activity Disorder Rating Scale IV; ASSQ, Autism Spectrum Screening Questionnaire; ATRS, Adelaide Teacher Rating Scale; CBCL, Child Behavior Checklist; CAI, Children's Aggression Inventory; CBI, Child's Behavior Inventory; CDI, Children's Depression Inventory; CHI, Conners' Hyperkinesis Index; CIS-R, Clinical Interview Schedule-Revised; YASR, Young Adult Self Report; CS, Connors scale; CSEI, Coopersmith Self Esteem Inventory; CMAS-R, Child Manifest Anxiety Scale-Revised; CAPA, Child and Adolescent Psychiatric Assessment; CSQ, Conners Symptoms Questionnaire; DSRS, Depression self-rating scale; HADS, the Hospital Anxiety and Depression Scale; MFQ, Mood and Feelings Questionnaire; OCHS-R, Ontario Child Health Study-Revised; RPQ, Rutter parent questionnaire; RTQ, Rutter teacher questionnaire; SAS, Social Activities Scale; SADSSAC, Schedule for Affective Disorders and Schizophrenia for School-Age Children; SDQ, Strengths and Difficulties Questionnaire; SF-36, Short Form 36 Health Survey; SPPA, the Self-Perception Profile for Adolescents; STAIC, State-Trait Anxiety Inventory for Children; TRF, Teacher Report Form; YSR, Youth Self-Report; TSCS, Tennessee Self-concept Scale; YABCL, Young Adult Behavioral Checklist*.

No consensus has yet been reached concerning the severity of emotional problems in preterm born adolescents (Nosarti et al., 2010) and the evidence for increased anxiety and depression is inconsistent (Johnson and Wolke, 2013). However, most case-control and birth cohort studies have described preterm adolescents as being emotionally vulnerable compared to controls. Significantly higher scores in preterm samples were reported using both the CBCL/SDQ emotional problems scales (Saigal et al., 2003; Gardner et al., 2004; Indredavik et al., 2005; Dahl et al., 2006; Farooqi et al., 2007) and specific tools assessing depression and anxiety (Botting et al., 1997; Saigal et al., 2003).

However, several other studies failed to detect significant emotional problems in VPT individuals (Rickards et al., 2001; Cooke, 2004; Grunau et al., 2004). A recent investigation by Hall and Wolke highlighted the need for longitudinal studies showing high stability of emotional problems and long lasting effects of child behavioral disturbances in a large cohort of VPT children from 6 to 13 years old (Hall and Wolke, 2012).

Methodological issues complicate the interpretation of results, as the type of “informer” used to collect information regarding emotional difficulties seems to affect findings. For instance, parents of VLBW adolescents reported more emotional difficulties in their offspring compared to parents of typically developing peers, whereas the VLBW adolescents in question declared less emotional problems than controls (Dahl et al., 2006).

Other possible confounding factors need to be taken into account when studying emotional difficulties in preterm adolescents: (1) symptoms of anxiety and depression show different life courses, with anxiety having a typical onset in childhood, compared to depression that mostly emerges in adolescence; (2) there are significant gender differences in prevalence of specific disorders, i.e., women are twice more likely to suffer from depression compared to men; (3) symptoms of anxiety and depression are highly comorbid, with anxiety often being a precursor to depression. Furthermore, in the preterm literature, anxiety and depression are often simultaneously investigated, which makes it difficult to disentangle the relative prevalence of dimension-specific symptoms (Johnson and Marlow, 2011).

### Socio-emotional behavioral problems in adulthood

There is emerging evidence of increased socio-emotional behavioral problems also among young adults who were born prematurely (Hack et al., 2004; Hille et al., 2008; Boyle et al., 2011; Lund et al., 2012; Saigal, 2014). Together with behavioral difficulties, personality traits of preterm born young adults have also been examined. Despite the use of different assessment methods and the investigation of geographically disparate cohorts, very preterm born individuals have quite consistently been described as being less extroverted, more cautious, shyer, and more risk aversive than controls (Allin et al., 2006; Hack, 2006; Pesonen et al., 2008; Schmidt et al., 2008; Hertz et al., 2013; Eryigit-Madzwamuse et al., 2015). Furthermore, all studies except the one by Pesonen et al. (2008) reported higher levels of neuroticism in preterm-born adults. These personality traits have been associated with an increased vulnerability to develop psychopathology (Kendler et al., 2006; Widiger, 2011; Hertz et al., 2013).

Recent investigations from the Nordic countries using population-based records have permitted large-scale studies of different aspects of preterm born adults' socio-emotional life. Even when free from major disabilities, preterm born adults are more likely to be living in the parental home. Norwegian, Swedish and Finnish cohort studies reported that preterm born adults were less likely to be married or cohabiting (Lindström et al., 2007; Kajantie et al., 2008; Moster et al., 2009), were less likely to have ever had sexual intercourse (Darlow et al., 2013) and reported fewer sexual partners compared to controls (Hack et al., 2002; Hille et al., 2008; Kajantie et al., 2008; Männistö et al., 2015). Although results are not conclusive, current evidence suggests that VPT individuals have a poorer social life, spend less time with friends, have fewer friends, have less confidence in romantic situations and tend to perceive themselves as less attractive (Lund et al., 2012; Saigal, 2014). These data support the idea that possible socio-emotional issues persist in preterm populations even during adulthood.

## Psychiatric disorders in very preterm born individuals

In addition to higher rates of behavioral problems, accumulating evidence supports an association between prematurity and increased risk of developing mental health problems. A recent meta-analysis revealed that preterm born children, adolescents and adults are about 3.5 times more likely to be diagnosed with a psychiatric disorder compared to term born peers (Burnett et al., 2011).

### Psychiatric disorders in childhood

Diagnostic studies describe a 3- to 4-fold risk of developing psychiatric disorders in preterm-born children compered to controls (Johnson and Marlow, 2011). Furthermore, preterm birth is associated with a specific pattern of disorders that mirrors the behavioral findings previously described: with preterm born children showing higher prevalence of ADHD (mainly the inattentive type), ASD and emotional disorders compared to their term born peers (Johnson and Marlow, 2011; Treyvaud et al., 2013). Published studies report a 2- to 3-fold risk for ADHD in VPT/VLBW children (Botting et al., 1997; Indredavik et al., 2004) and a 4-fold increased risk in extremely preterm (EPT; < 26 weeks of gestation) and ELBW children (Hack et al., 2009; Johnson et al., 2010b). The inattentive ADHD subtype seems to be prevalent and the clinical presentation of the disorder is quite different from that observed in children with ADHD who were not born VPT/VLBW, as it is associated with neurocognitive impairments and social difficulties, such as shyness, withdrawal and internalizing problems (Diamond, 2005).

Autism spectrum disorder has been reported as being 4–12 times more prevalent among ex-preterm children compared to controls (Williams et al., 2006; Fombonne, 2009) with symptoms believed to be qualitatively different from classical autistic traits, in this case being mediated by cognitive impairments, including distractibility and inattention (Hille et al., 2001; Elgen et al., 2002). Moreover, in contrast to the characteristic larger head circumference found in ASD children, in preterm children ASD symptoms have been found to be significantly associated with smaller head circumference (Johnson et al., 2010a).

Together with ADHD and ASD, diagnostic studies describe an increased prevalence of emotional disorders following VPT/VLBW, indicating a specific risk for anxiety in childhood. In contrast to the general population, emotional disorders do not show a female prevalence and seem associated with cognitive impairments (Johnson and Marlow, 2011).

### Psychiatric disorders in adolescence and adulthood

While diagnostic studies in preterm children mirror the profile of impairments described by a “preterm behavioral phenotype,” in adolescence and young adulthood prematurity has been defined as a single independent risk factor for a wide range of psychiatric disorders (Nosarti et al., 2012).

Different study designs have complementarily been used to investigate the prevalence of psychiatric disorders in preterm individuals. On the one hand, register studies in Scandinavian countries have provided a fruitful source of information using population-wide records. These studies have shown a stepwise increase in hospital admissions with decreasing gestational age (Lindström et al., 2007), and an increased risk of receiving a psychiatric diagnosis (Abel et al., 2010) and pharmacological treatments (Crump et al., 2010) in preterm born individuals compared to term-born peers. A higher risk of anorexia nervosa has been further reported in VPT girls (Cnattingius et al., 1999), as well as an increased prevalence of ADHD and ASD in VPT adults (Moster et al., 2009; Halmøy et al., 2012). These results suggest that several psychiatric outcomes may be associated with the same risk factor and a recent population-based study of 1,300,000 individuals demonstrated that VPT birth conferred an increased risk of hospitalization for non-affective psychosis, depressive and bipolar disorder, independently of other neonatal risk factors (Nosarti et al., 2012). Such findings suggest the existence of similar developmental mechanisms linking various psychiatric disorders, an idea which is supported by the results of family studies showing an increased risk of a variety of psychiatric disorders, including those not considered as being clinically associated, in individuals with a psychiatric family history (Dean et al., 2010).

Although these data from population-wide studies are informative, conditions that do not require pharmacological intervention or hospitalization, such as anxiety or mood disorders, are not recorded into register studies and alternative sources of information should be also pursued in order to gain diagnostic details. Clinical case-control studies employing psychopathology questionnaires reported an increased risk of mood and anxiety disorders associated with premature birth (Botting et al., 1997; Elgen et al., 2002; Indredavik et al., 2004; Walshe et al., 2008; Johnson et al., 2010a; see Burnett et al., 2011 for review), with several birth cohort studies suggesting almost a 2-fold risk of anxiety problems in VPT/VLBW populations (9.9% vs. 5.5% prevalence; Sømhovd et al., 2012), as well as a significant association between prematurity, intrauterine growth and depression (Thompson et al., 2001; Gale and Martyn, 2004; Alati et al., 2007; Raikkonen et al., 2008).

## Brain correlates of socio-emotional and mental health problems

Converging evidence shows that survivors of very preterm birth are at substantial risk of brain injury in the perinatal period (Volpe, 2009). Primary focal lesions of the immature brain occur during a period of rapid development (for instance, the brain roughly triples in weight during the third trimester of gestation), and such lesions may disrupt programmed corticogenesis (Volpe, 2009) and subsequent typical maturational processes by altering cortical and subcortical developmental patterns (Hack and Taylor, 2000).

Given the association between prematurity and altered neurodevelopment (Ball et al., 2013), specific structural and functional brain alterations may underlie the socio-emotional difficulties associated with VPT birth. A huge amount and variety of mental operations are devoted to social information and emotion processing (Happé and Frith, 2014), and anatomically distributed networks have been shown to be part of a so-called social brain (Blakemore, 2008). These networks involve regions in the temporal lobe for processing faces, amygdala and insula for detecting and responding empathetically to others' emotions, orbitofrontal areas for emotional evaluation, threat detection and emotion regulation, medial prefrontal areas and superior temporal regions for the automatic attribution of mental states, while parietal and prefrontal areas have been implicated in processing other people's actions (Kennedy and Adolphs, 2012).

**Figure 1:**
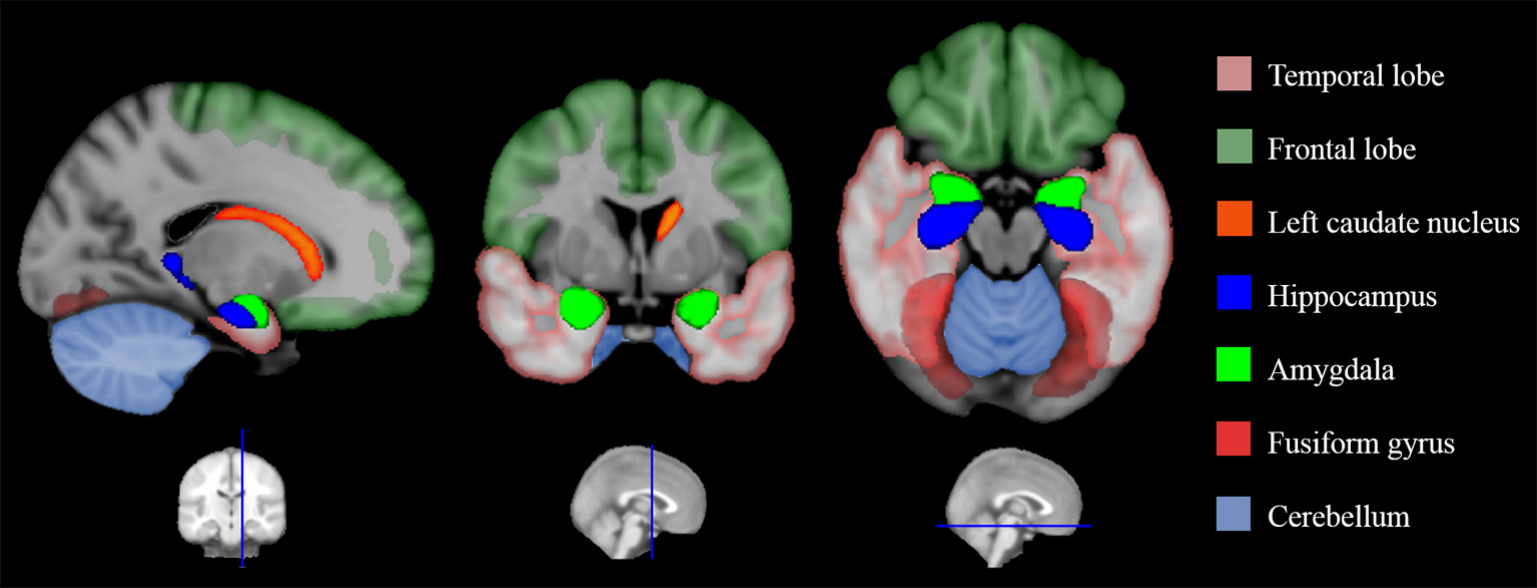
**Structural alterations in the social brain found in preterm individuals**. This is a graphic representation of a summary of the studies reviewed in Table 3. Colored areas highlight brain regions involved in processing socio-emotional stimuli that have been shown to display structural alterations in very preterm samples.

Volumetric alterations in some of these areas have been shown in VPT samples in childhood and adolescence: decreased gray matter concentration in orbitofrontal cortex (Gimenez et al., 2006; please see Ganella et al., 2015 for differences in orbitofrontal cortex sulcogyral pattern in EPT/ELBW adolescents), reduced volume of fusiform gyrus (Nosarti et al., 2008; Gousias et al., 2012), amygdala (Peterson et al., 2000), insula (Nosarti et al., 2008, 2014), and hippocampus (Nosarti et al., 2002; Abernethy et al., 2004; Rogers et al., 2012; Omizzolo et al., 2014; Aanes et al., 2015; Figure 1).

Only a few studies to date have investigated socio-emotional outcomes following preterm birth in association with structural and functional brain alterations (Nosarti et al., 2014; please refer to Table 3 for a list of studies associating MRI findings with socio-emotional outcomes).

**Table 3:** **MRI studies in relation to socio-emotional behavioral outcomes in VPT samples**.

**Author**	**Type of study**	**Methods**	**Cases (n)**	**Controls (n)**	**Age (y)**	**Outcome measure of socio-emotional behavioral functioning**	**Results**
Clark et al., 2008	Longitudinal with imaging at term equivalent age	MRI at 40 weeks post-conception, 2 longitudinal assessments.	39 (<28 w) 55 (<34 w)	103	2, 4	At the two time points: -Observed child behavior during parent-child interaction. -Behavior rating completed after cognitive testing. -ERC	Qualitatively assessed WM alterations detected at the time of the expected date of delivery have been shown to be associated with poorer emotion regulation in the group of preterm toddlers.
Fischi-Gómez et al., 2015	Cross-sectional	Structural Connectome	20 (<28 w) 17 (33-37 with IUGR)	8 (33–37 w)	6	SDQ	Connections between medial orbito-frontal, prefrontal, parietal cortex, and the basal ganglia are correlated with SDQ prosocial subscale scores in children born moderately premature with intrauterine growth restriction.
Ganella et al., 2015	Cross-sectional	MRI and interviews at 18 years. Analysis of orbitofrontal cortex sulcogyral pattern.	194 (<28 w)	147	18	CIPS SCID-IV	Type II orbitofrontal pattern was more frequent in the EPT group (only in the left hemisphere). Differences in sulcogyral patterns were not associated with mental health disorders.
Healy et al., 2013	Longitudinal during adolescence	MRI at 15 years and questionnaires at two time points (15 and 19 years).	73 (<33 w)	49	15, 19	CBCL social scale CIS-R	Increased bilateral gray matter volume in the fusiform gyrus in socially immature VPT adolescents at 15. Increased left fusiform volume correlated with ipsilateral orbitofrontal cortex volume. Atypical social development at 15 predicted higher vulnerability to psychiatric disorders at 19.
Jones et al., 2013	Longitudinal with imaging at term equivalent age	MRI at 40 weeks post-conception and developmental assessment.	103 (≤32 w)	105 (36–41 w)	4	SDQ BRIEF-P, inhibitory control subscale ERC ITSC, self-regulation subscale. PIPPS	VPT children showed poorer emotional and behavioral adjustment, were less effective in regulating their emotions, had lower levels of positive peer play and had less synchronous interactions with their parents. Increasing severity of cerebral white matter abnormalities on term MRI (qualitative assessment) was associated with increased risk of poorer social competence at age 4 years for VPT children.
Limperopoulos et al., 2007	Longitudinal with imaging at term equivalent age	MRI at 40 weeks post-conception and developmental assessment.	35 (<32 w with isolated cerebellar haemorrhagic injury) 16 (with cerebellar haemorrhagic injury plus supratentorial parenchymal injury)	35	32 months	ASQ	Specific increased risk for internalizing behaviors and autism-like symptoms in preterm born children with perinatal cerebellar haemorrhagic injury.
Loe et al., 2013	Cross-sectional	Diffusion tensor imaging	25 (<36 w)	20	9-16	CBCL	Lower FA (a measure of white matter microstructural alterations) in a series of white matter (WM) tracts (forceps major, forceps minor, inferior fronto-occipital fasciculus/inferior longitudinal fasciculus, superior longitudinal fasciculus, and corticospinal tract) was associated with increased attention and internalizing problems in a sample of PT children.
Nosarti et al., 2005	Cross-sectional	Structural MRI—ROI us	66 (<33 w)	50	14–15	CSQ	Left caudate volume was negatively correlated with social adjustment score in VPT adolescents.
Nosarti et al., 2011	Longitudinal with imaging at term equivalent age	Neonatal cranial ultrasound (US) and neuropsychological tests and questionnaires at 14–15 years.	120 (<33 w divided in 3 groups: 69 with normal US, 37 with PVH 14 with PVH and DIL)	50	14–15	RPS PAS	A predictive role of neonatal ultrasound abnormalities on behavioral and social adjustment outcomes. Significant increase in generalized behavioral problems and social adjustment issues in a VPT born adolescents with a history of periventricular hemorrhage and ventricular dilatation.
Parker et al., 2008	Longitudinal during adolescence	MRI and questionnaires at two time points (15 and 19 years).	65 (<33 w)	34	15, 19	GHQ	Cerebellar volumetric changes between VPT subjects and controls in the transition from adolescence to young adulthood (15–19 years). Between the two time-points, the VPT group showed a cerebellar shrinkage of 3% and this decrease was associated with worse mental health as assessed by the self-report.
Rogers et al., 2012	Longitudinal with imaging at term equivalent age	MRI (diffusion tensor imaging) at 40 weeks post-conception and developmental assessment at 2 and 5 years old.	111 (<30 w)	0	2, 5	ITSEA SDQ	Higher apparent diffusion coefficient (ADC) in the right orbitofrontal cortex detected at term equivalent age was related to peer problems assessed with SDQ in VPT children. SDQ peer problems, hyperactivity and total scores were correlated with smaller hippocampal volume in females, while SDQ poorer prosocial scores were associated with a smaller frontal region in boys.
Rogers et al., 2014	Cross-sectional	Structural MRI—ROI	21 (34–36 w)	87 (40–41 w)	6–12	PAPA for children < 6. CAPA for children >6.	Reduced volume of the right temporal lobe was related to later anxiety symptoms in the group of late preterm children.
Schmidt et al., 2010	Longitudinal with imaging at term equivalent age	Frontal Electroencephalogram (EEG) Asymmetry	71 (<1000 grams)	83	23	Measure of relative frontal EEG asymmetry	Internalizing problems in young adults who were born with an extremely birth weight were associated to greater relative right frontal EEG activity.
Skranes et al., 2007	Cross-sectional	Diffusion tensor imaging	34 (≤1500 grams)	47	15	ASSQ CGAS	White matter microstructural alterations in the superior fasciculus and external capsule were associated with high scores on an autism spectrum screening in VLBW adolescents
Spittle et al., 2009	Longitudinal with imaging at term equivalent age	MRI at 40 weeks post-conception and developmental assessment	188 (<30 w)	70	2	ITSEA	White matter abnormalities detected on term MRI were associated with behavioral problems (internalizing and dysregulation problems) at 2 years in the VPT group.
Stewart et al., 1999	Longitudinal with imaging at term equivalent age	Structural MRI	72 (<33 w)	21	14–15	RBS	Abnormal MRI findings detected at 14/15 years old were associated with behavioral problems and difficulties in social adjustment (Premorbid Adjustment Scale) in very preterm adolescents.
Zubiaurre-Elorza et al., 2012	Cross-sectional	Structural MRI—Cortical Thickness	22 (<37 w, with history of PVL) 14 (<37 w, without history of PVL)	22	6–12	CBCL	Internalizing and externalizing problems (CBCL) were associated with thinner frontal cortical thickness in preterm born children with history of periventricular leukomalacia

*ASSQ, Autism Spectrum Screening Questionnaire; BRIEF-P, Behavioral Rating Inventory of Executive Function—Preschool version; CAPA, Child and Adolescent Psychiatric Assessment for children >6; CBCL, Child Behavior Checklist; CIS-R, Clinical Interview Schedule-Revised; CSQ, Cannon-Spoor questionnaire; CGAS, Children's Global Assessment Scale; ERC, Emotion Regulation Checklist; GHQ, General Health Questionnaire; ITSC, Infant–Toddler Symptom Checklist; ITSEA, Infant Toddler Socio-Emotional Assessment; PAPA, Preschool-Age Psychiatric Assessment for children < 6; PAS, the Premorbid Adjustment Scale; PIPPS, Penn Interactive Peer Play Scale; RBS, Rutter Behavioral Scale; RPS, Rutter Parents Scale; SDQ, Strengths and Difficulties Questionnaire*.

Results of neuroimaging studies looking at defined cross-sectional time points showed significant associations between smaller volume of left caudate nucleus (Nosarti et al., 2005) and right superior temporal lobe (Rogers et al., 2014) and social adjustment and anxiety problems, respectively. Caudate abnormalities were also described in ASD (Qiu et al., 2010), suggesting a role of the caudate nucleus in reciprocal social and communicative behavior, possibly due to its complex connections within cortical-basal ganglia circuits underlying emotional and cognitive habits (Arnsten and Rubia, 2012). Structural brain alterations in temporal cortex have been described as core long-term consequences of VPT (Nosarti et al., 2008, 2014). Furthermore, alterations of the superior temporal lobe have been specifically associated with anxiety disorders (Corbetta et al., 2008).

Internalizing and externalizing problems were also associated with thinner prefrontal cortices in VPT children (Zubiaurre-Elorza et al., 2012) providing further support for the importance of frontal cortex maturation for appropriate behavioral control and inhibition (Shaw et al., 2012). Another aspect of brain function that has been related to internalizing problems in ELBW young adults was greater relative right frontal electroencephalography (EEG) activity. This measure is thought to be linked to the processing of negative emotions and has been suggested as a possible mechanism predisposing individuals to experience problems in stress regulation (Schmidt et al., 2010).

The association between socio-emotional problems and gray matter in the “social brain” were studied by Healy et al. (2013), who found that socially immature VPT adolescents (i.e., those with worse scores on the CBCL social problems scale) had increased bilateral gray matter volume in the fusiform gyrus compared to their VPT peers with age-appropriate CBCL scores, suggesting neurodevelopmental delays. Socio-emotional problems have been also associated with alterations in white matter (WM) areas that connect different brain regions involved in the processing of social stimuli and in WM tracts known to play a role in attention and working memory. Microstructural alterations in the superior fasciculus and external capsule were associated with high scores on an autism spectrum screening in VLBW adolescents (Skranes et al., 2007), while microstructural alterations in several WM tracts (forceps major, forceps minor, inferior fronto-occipital fasciculus/inferior longitudinal fascisculus, superior longitudinal fasciculus, and corticospinal tract) were associated with increased attention and internalizing problems in preterm children (Loe et al., 2013). Another recent study further described significant associations between WM connectivity in medial orbito-frontal, prefrontal, parietal cortex, and the basal ganglia and SDQ prosocial subscale scores in 6 years old children born moderately preterm (32 to < 37 weeks of gestation) with intrauterine growth restriction (Fischi-Gómez et al., 2015).

These studies highlight the complex interplay among different brain structures and the role of their connectivity in maintaining unimpaired social cognition and social behaviors. Fronto-striato-cerebellar circuits in addition to fronto-limbic and fronto-parietal networks seem to contribute to optimal socio-emotional behaviors. The “social brain,” far from being a modular and anatomically defined set of brain areas, appears to rely on distributed circuits, with the processing of social stimuli requiring the coordinated action of systems regulating attention, cognitive control, motivation, emotion and social cognition.

Structural and functional brain alterations associated with VPT birth in the early stages of development may affect later development of these networks. Results of the few longitudinal studies conducted to date have reported a significant association between perinatal white matter alterations and socio-emotional outcomes in childhood, pointing to the possibility of identifying possible biomarkers of outcome long before the actual manifestation of any potential problem (Clark et al., 2008; Spittle et al., 2009; Jones et al., 2013).

Higher apparent diffusion coefficient (ADC), reflecting possible WM pathology, in right orbitofrontal cortex detected at term equivalent age in VPT infants was related to peer problems at 5 years, supporting the idea of the involvement of the orbitofrontal cortex in theory of mind, social cognition, and social regulation (Rogers et al., 2012). The same study also described gender differences in the association between socio-emotional development at age 5 and regional brain maturation at term: SDQ peer problems, hyperactivity and total scores were correlated with smaller hippocampal volume in females, while poorer SDQ prosocial scores were associated with a smaller frontal region in boys. Another study described a specific increased risk for internalizing behaviors and autism-like symptoms in preterm born children with perinatal cerebellar haemorrhagic injury, highlighting the role of cerebellum in behavioral and social dysfunctions (Limperopoulos et al., 2007). Together with these finding, Nosarti et al. (2011) showed a predictive role of neonatal ultrasound abnormalities on behavioral and social adjustment outcomes in adolescence. They described a significant increase in generalized behavioral problems and social adjustment issues in a VPT born adolescents with a history of periventricular hemorrhage and ventricular dilatation, highlighting the importance of the integrity of subcortical periventricular areas for the optimal development of networks underlying socio-emotional functioning.

Only one study to date has investigated mental health and socio-emotional development in VPT samples in association with volumetric changes beyond the first weeks of life. Parker et al. (2008) compared cerebellar volumetric changes between VPT individuals and controls during the transition from adolescence to young adulthood (14–19 years). Between the two time-points, the VPT group showed 3% cerebellar shrinkage, and this decrease was associated with worse mental health, as assessed by a self-report (the General Health Questionnaire). This study highlights the need and importance of longitudinal investigations to shed light on the dynamic patterns of cortical and subcortical maturation associated with socio-emotional development and psychiatric outcomes (Shaw et al., 2010).

## Pathways to psychopathology

Despite a great prevalence of socio-emotional and psychiatric problems in individuals born very preterm, the underlying mechanisms that lead from preterm birth to socio-emotional vulnerability and psychopathology are far from understood. The hypothesis that socio-emotional difficulties lay on the causal pathway to developing psychiatric disorder has been supported by a great wealth of studies and socio-emotional problems have been considered as an important risk factor for psychopathology (Carter et al., 2004).

According to the social defeat hypothesis of mental illness, successful social functioning, including adequate social and emotional support, represents a protective factor against the development of psychiatric disorder (Selten and Cantor-Graae, 2005). Chronic experience of psychosocial stress and social adversity often results in social marginalization and feelings of inferiority, and these in turn increase an individual's risk for psychopathology. This conceptualization finds support in animal studies (see the “intruder-resident paradigm”) and in epidemiological human research, which shows a higher incidence of psychiatric outcomes in populations experiencing high social stress, such as migrants and individuals raised in urban environments (Van Os et al., 2010).

Dopamine dysregulation has been put forward as representing the possible biological mechanism linking social defeat with an increased risk of psychopathology: long-term exposure to social defeat may lead to sensitization of the mesolimbic dopamine system, to increases in its baseline activity, and thereby to a greater risk for psychosis (Selten and Cantor-Graae, 2005). Moreover, socio-emotional difficulties, such as affective dysregulation (as well as alterations in dopaminergic function), have been described as features characterizing the “extended phenotype,” and as a behavioral expression of vulnerability for psychiatric disorders (Van Os and Linscott, 2012).

Despite current evidence linking socio-emotional vulnerability with an increased risk of developing psychopathology, to date only a few studies have investigated the etiology of socio-emotional risk in VPT samples. The following section will offer an overview of several possible mechanisms which have been put forward in order to understand socio-emotional vulnerability and its antecedents.

### Socio-emotional problems as a result of impaired social competence and their neural substrates

The term “social competence” refers to a variety of mental mechanisms aimed at supporting successful social functioning, including emotional self-regulation, social cognitive processing, positive communication and prosocial social relationships (Bornstein et al., 2010; Jones et al., 2013). Specific brain networks have been found to subserve these processes and to form the so-called “social brain” (Kennedy and Adolphs, 2012). Cross-sectional and longitudinal investigations of the associations between social competence and socio-emotional behavioral adjustment abound in the literature (Masten et al., 2006; Parker et al., 2006; Rubin et al., 2006). Overall, results of such studies suggest that deficits in social competence detected early in life contribute to the formation of internalizing problems, which may develop later on.

In light of these studies, several authors support a sociobiological vulnerability approach, which postulates that the increased social vulnerability seen in VPT samples occurs as a result of specific alterations in the “social brain,” as part of the neurodevelopmental sequelae of VPT birth (Healy et al., 2013). However, although social competence, achieved in the context of its typically developing neural substrates, has been recognized as the cornerstone of successful social adjustment, to date only a few studies have systematically investigated the early development of social competence in children born preterm. Furthermore, there is a paucity of investigations that have comprehensively assessed core aspects of development known to represent the milestones of successful social functioning.

#### Early-life joint attention

Joint attention skills have been described as important precursors of social development (Baron-Cohen, 1991; Carpenter et al., 1998; Mundy and Gomes, 1998) and have been extensively investigated in the context of ASD research. Studies by Landry (1986) reported difficulties in joint attention in 6 months old infants born very preterm: these infants showed more problems engaging in joint play and initiating joint-attention interactions with their mothers, and exhibited more gaze aversion compared to their term-born peers (Garner et al., 1991; Smith and Ulvund, 2003).

#### Emotion recognition

Four studies to date have investigated the ability to recognize facial emotional expressions in preterm born individuals. Recognizing facial emotional expressions is fundamental for developing effective social interactions and social adjustment (Saarni, 1999), and deficits in emotional understanding are associated with socio-emotional problems and psychiatric disorders (Denham et al., 2003; Dunn, 2010). Difficulties in emotion recognition were described in preterm children: Witt et al. (2014) reported less accurate naming of facial expressions of emotions in a sample of 42 months old VPT children compared to controls, while Potharst et al. (2013) reported similar finding in VPT children aged 5. Wocadlo and Rieger (2006) provided comparable results in a sample of 8 year olds and confirmed the role of emotion recognition for successful social functioning, showing an association between impaired emotion recognition, less popularity and more negative nominations from peers (Wocadlo and Rieger, 2006). Using a more complex test of social perceptual skills, Williamson and Jakobson (2014a) also described problems in interpreting others' emotions in 8–11 years old VPT children.

#### Emotion regulation

Emotion regulation refers to a child's ability to modulate his/her emotions in response to people and situations, using a range of cognitive, physiological and behavioral processes/strategies allowing for empathic and socially appropriate behavior. Emotion regulation has increasingly been recognized as a potentially crucial marker of later psychosocial risk (Cole et al., 1994; Lawson and Ruff, 2004). Emotion regulation was longitudinally tested in a group of VPT children at 2 and 4 years using three different measures, consisting of two questionnaires (the emotion-regulation subscale from the Emotion Regulation Checklist and the Infant–Toddler Symptom Checklist) and in observational ratings of the child's emotion regulation during a session of developmental testing. Higher mean levels of emotional dysregulation emerged at both time points in the VPT group compared to controls (Clark et al., 2008; Jones et al., 2013).

#### Social cognition

The term “social cognition” refers to the fundamental abilities to perceive, store, analyze, process, categorize, reason with, and behave toward other conspecifics (Pelphrey and Carter, 2008). Biological motion perception and theory of mind reasoning are two essential aspects of social cognition. Biological motion perception is defined as the ability to perceive and interpret body movements and is considered as a hallmark of social cognition (Pavlova, 2012). Theory of mind is defined as the ability to understand that other people may have different motivations and emotions from one's own and that people's behavior is guided by their inner states (Carlson and Moses, 2001). Impaired theory of mind has been described as a core deficit in autism spectrum disorder and has been linked to social anxiety and low popularity with peers (Happé and Frith, 2014).

Only a few studies to date have investigated these aspects of social cognition in preterm populations, and overall results suggest suboptimal functioning in both. VPT born children were less able to process biological motion and to attribute mental states than their term-born peers (Pavlova et al., 2008; Taylor et al., 2009; Williamson and Jakobson, 2014a,b; in VPT children with periventricular leukomalacia). However, no significant impairments in theory of mind tests were found by Jones et al. (2013) in a sample of VPT children aged 4, although the authors suggested the need for further investigations, as the psychometric utility of traditional false belief tests used in this study has been questioned.

#### Social competence investigated using questionnaires

Two studies to date have investigated social competence using the social competence scale of the Infant Toddler Socio-Emotional Assessment (ITSEA) and the Vineland scales (Sparrow et al., 1989), respectively. In both studies, VPT born children aged 2–5 scored significantly lower than their term born peers (Spittle et al., 2009; Alduncin et al., 2014).

Further research is needed to examine the presence of specific impairments in social cognition, emotion regulation, communicative skills and interactive behaviors in preterm children. More research is also required to shed light on possible alterations in patterns of neurodevelopment associated with impaired social competence, as no study thus far has targeted the possible neural underpinning of impaired social competence in preterm populations. A growing area of interest is focusing on the identification of early markers of social impairments, as studies have shown a predictive role of early abnormal patterns of attention orienting and eye gazing in discriminating individuals at risk for impaired social competence (Wass et al., 2015).

### Socio-emotional problems resulting from cognitive and motor impairments

The previously described socio-biological vulnerability model explains socio-emotional difficulties resulting from possible structural and functional brain alterations in the “social brain” and postulates that, in preterm born individuals, socio-emotional brain networks and associated mental processes show atypical neurodevelopmental trajectories. These specific socio-biological alterations are thought to be due to preterm birth itself and to the range of possible clinically adverse events associated with it. In contrast to this model, several authors advocate for a non-specific etiology of socio-emotional difficulties in preterm populations, suggesting that cognitive impairments may mediate the association between preterm birth and socio-emotional difficulties. It is in fact well known that preterm birth represents a risk factor for a variety of neurodevelopmental sequelae and that impaired general cognitive functions (IQ) is a common finding. Convincing evidence exists to suggest that overall cognitive ability is reduced in preterm populations, and a recent meta-analysis describes a gradient effect of VPT birth on IQ, with an average estimated decline of 1.5 IQ points for each weekly decrease in gestational age for those born < 33 weeks of gestation (Kerr-Wilson et al., 2012; Anderson, 2014).

The hypothesis of a non-specific cognitive origin of socio-emotional behavioral problems explains the pattern of behavioral problems observed in VPT samples by hypothesizing a role of perinatal diffuse white matter injury in both cognitive and socio-emotional outcomes. In this model, the biological vulnerabilities associated with prematurity are thought not to be limited to areas involved in social information processing, but to affect several networks causing widespread functional impairments. This hypothesis brings attention to the role of thalamocortical connections, which are among the most severely damaged structures after preterm birth (Volpe, 2009; Kostović and Judaš, 2010; Ball et al., 2013) and play a crucial role in brain development (Kostović and Jovanov-Milošević, 2006), adversely affecting the maturation of several cortical and subcortical brain regions throughout the brain (Hack and Taylor, 2000; Ball et al., 2013). Moreover, altered thalamocortical connectivity in preterm infants has been found to predict general cognitive functions at 2 years of age (Ball et al., 2015).

The hypothesis of a cognitive root of socio-emotional difficulties comes from research investigating multiple predictors of behavior in the general population, as well as in preterm individuals (Goodman et al., 1995). Several studies have shown a co-occurrence of cognitive and socio-emotional difficulties, highlighting the important contribution of cognitive abilities to interpersonal skills and social maturity (Hughes et al., 2000; Stuss and Alexander, 2000; Heiervang et al., 2001; Kuntsi et al., 2001; Clark et al., 2002). Moreover, low IQ is one of the characteristic features of the extended phenotype and a core marker of vulnerability to psychopathology (Van Os and Linscott, 2012).

A possible pathway through which cognition influences behavioral problems may be through its effect on academic achievement: cognitive and learning difficulties may affect school performance, and academic underperformance may in turn contribute to children's psychosocial problems, posing risks for victimization and social exclusion (Gadeyne et al., 2004; Nadeau et al., 2004).

The model of socio-emotional difficulties following preterm birth is supported by a series of studies which found that cognitive abilities explained the observed increase in socio-emotional difficulties in preterm born samples. Mansson et al. (2014) showed a mediating effect of the mental developmental index (MDI) on internalizing problems in a sample of EPT toddlers. In line with these findings, in a sample of VPT born 8 year olds, the association between birth status, emotional symptoms and poorer social skills was mediated by IQ (Bayless et al., 2008).

Similar findings emerged from diagnostic studies: Johnson et al. (2010a), found that cognitive impairment accounted for more than half of the excess of socio-communication problems in 10 year olds born EPT with a diagnosis of autism, while in another cohort of 11 years old EPT children cognitively impaired children accounted for a significant portion of the diagnosed emotional disorders (Johnson et al., 2010b).

Another factor thought to mediate socio-emotional difficulties in preterm populations is neuromotor impairment. Up to 40% of children born very preterm suffer minor motor problems (Kieviet Piek et al., 2009) and motor difficulties have been shown to have far-reaching implications for socio-emotional function, as they have been linked with low self-esteem, difficulties in peer relations, isolation and emotional disorders (Skinner and Piek, 2001). In a study by Nadeau et al. (2001), neuromotor problems were found to explain sensitive and isolated behaviors in EPT born 5-years-olds.

However, despite the findings of studies summarized above, showing a consistent association between cognitive impairment and socio-emotional vulnerability in VPT samples, in the majority of cases, cognitive and motor impairments alone do not account for the excess of socio-emotional problems seen in VPT individuals, and further investigation of the etiology of such problems is required (Johnson and Marlow, 2014).

### Socio-emotional problems result from environmental factors—early life stress and parenting

Other possible mechanisms that could be studied in order to achieve a greater understanding of the etiology of socio-emotional problems in VPT individuals are environmental factors thought to interact throughout child development. Two principal factors have been described in the preterm literature, namely painful experiences in the perinatal period and parental stress in the early stages of life.

#### Perinatal pain exposure and socio-emotional vulnerability

Together with an increased risk of medical complications, very preterm birth exposes infants to prolonged and repeated pain-related procedures as part of their care in the neonatal intensive care unit. Neonatal pain is quantified as the number of skin-breaking procedures from birth to term equivalent age and has been found to be associated with altered stress hormone (cortisol) regulation and lower motor and cognitive functions at 8 and 18 months corrected age (Grunau et al., 2009). Greater pain-related stress has been recently associated with greater internalizing (anxiety and depression) behaviors at 18 months corrected age (Vinall et al., 2013) and 7 years (Ranger et al., 2014).

Although the role of neonatal pain in the etiology of internalizing behavioral difficulties is unknown, these new findings are in line with results of animal studies which showed that rat pups exposed to neonatal pain exhibited more anxiety-mediated behaviors in adulthood than control animals (Anand et al., 1999). Moreover, recent studies have demonstrated substantial effects of neonatal pain on the vulnerable developing brain, triggering both oxidative stress and inflammatory reactions, affecting the development of preoligodendrocytes and subplate neurons due to the excessive release of glutamate and influxes of calcium (Vinall and Grunau, 2014).

Neonatal pain has been associated with altered white matter microstructure and subcortical delayed gray matter maturation (Smith et al., 2011; Brummelte et al., 2012). These findings are particularly relevant in light of recent studies reporting a predicting role of brain alterations detected in the neonatal period for later socio-emotional behavioral problems (see the Section Brain Correlates of Socio-Emotional and Mental Health Problems).

#### Parental stress in the early stages of life

Parental behavior plays a crucial role in the early stages of their child's life, as developmental vulnerability is associated with parents' ability to buffer against high-risk events.

An example of the protective role of parenting is found in the studies described above: among preterm children exposed to greater pain procedures, parental sensitivity and non-hostility were shown to predict reduced internalizing behaviors at both 18 months and 7 years (Vinall et al., 2013; Ranger et al., 2014). Several other studies provided strong evidence for the role of parenting in protecting against early life stress, and in population-based surveys, parenting style and parental mental health (especially depression and anxiety) were shown to modulate socio-emotional development. Maternal distress, in particular, was associated with children's behavioral difficulties and this association held true for VPT children (Treyvaud, 2014).

Preterm birth and hospitalization are highly stressful experiences for parents. Preterm delivery interrupts the normal process of becoming a parent and parenting distress seems to persist long beyond hospital discharge, with parents showing ongoing concerns about their child's health and development. Parents of children born very preterm have been described as having more psychological distress (Huhtala et al., 2011, 2012, 2014; Schappin et al., 2013), depression (Silverstein et al., 2010; Vigod et al., 2010), and post-traumatic stress symptoms (Pierrehumbert et al., 2003; Kersting et al., 2004; Ahlund et al., 2009) than parents of term-born children. Although limited evidence is available regarding the stability of these symptoms, parental functioning seems to improve with time, with the first period of the child's life appearing as the “at-risk” time window for parental mental health (Treyvaud, 2014).

Maternal depression and parental stress may modify a mother's adjustment to her child, her perceptions, attitudes and parenting styles, thereby impacting on the child's socio-emotional outcomes. In support of this hypothesis, parental stress was shown to act as an independent predictor of internalizing behaviors in VLBW 2 year olds (Zelkowitz et al., 2011). Other evidence comes from the investigations of Huhtala et al. (2011, 2012, 2014) who described a significant association between poorer parental mental health and emotional behavioral problems in VLBW 2, 3, and 5 year olds. Similar results were described by Treyvaud et al. (2010, 2012): parental mental health and a more optimal home environment were associated with better socio-emotional regulation in VPT toddlers aged 2 years. Similarly, lower levels of maternal anxiety and intrusivity predicted better social skills also in VPT 4 year olds (Jones et al., 2013). Finally, McCormick et al. (1996) described a negative association between emotional well-being and behavioral problems and maternal mental health in VLBW school aged children.

Maternal depression was found to impact children's social abilities: Silverstein et al. (2010) described an association between maternal depression, negative perceptions of their child's social skills and reduced age appropriate behaviors in a sample of VPT toddlers. Maternal distress was also found to predict internalizing problems and social skills in samples of 36 and 48 months old children, respectively (Miceli et al., 2000; Assel et al., 2002).

These studies highlight the role of parental mental health on a child's socio-emotional development, although not much is known regarding the mechanisms through which parental status may affect different trajectories of socio-emotional development. According to the studies illustrated at the beginning of the section, a possible mechanism could involve a mediating role of parenting on the effect of early stressful adverse life events (Vinall et al., 2013; Ranger et al., 2014), possibly in scaffolding the child's development of self-regulation (Rueda et al., 2004).

Despite this, it is worth noticing that parenting itself is influenced by both children's and parents' characteristics. Therefore, the causal direction of the association between socio-emotional behavioral problems in childhood and parenting is difficult to establish (Treyvaud, 2014).

## Developmental mechanisms underlying the association between preterm birth, socio-emotional difficulties and psychopathology

According to the studies reviewed here, several factors can contribute to the understanding of the association between preterm birth, socio-emotional difficulties and psychopathology. However, further investigation is required to shed light on the mechanisms through which these factors dynamically interplay during development in preterm born samples. In this paper we focused on the description of socio-biological vulnerabilities, neurocognitive/motor deficits and environmental influences as possible factors linking preterm birth and an increased vulnerability to socio-emotional behavioral problems.

Although such factors have been independently associated with the type of socio-emotional problems seen in preterm populations, only a few studies to date have analytically investigated their interactions during the course of development. According to a bio-ecological, transactional, developmental framework (Sameroff and Chandler, 1975; Bronfenbrenner, 1986; Lerner, 1991; Sameroff, 1995), various aspects of development including neural maturation, psycho-social functioning, familiar and cultural environment, dynamically interact in the first years of life. Within this framework, preterm birth concomitantly affects all these aspects of development, precipitating a cascade of neurodevelopmental outcomes which affect the individual, together with the entire family and extended social systems. According to this model, biological vulnerabilities and environmental factors are believed to interdependently interact and contribute to socio-emotional and psychiatric outcomes.

**Figure 2:**
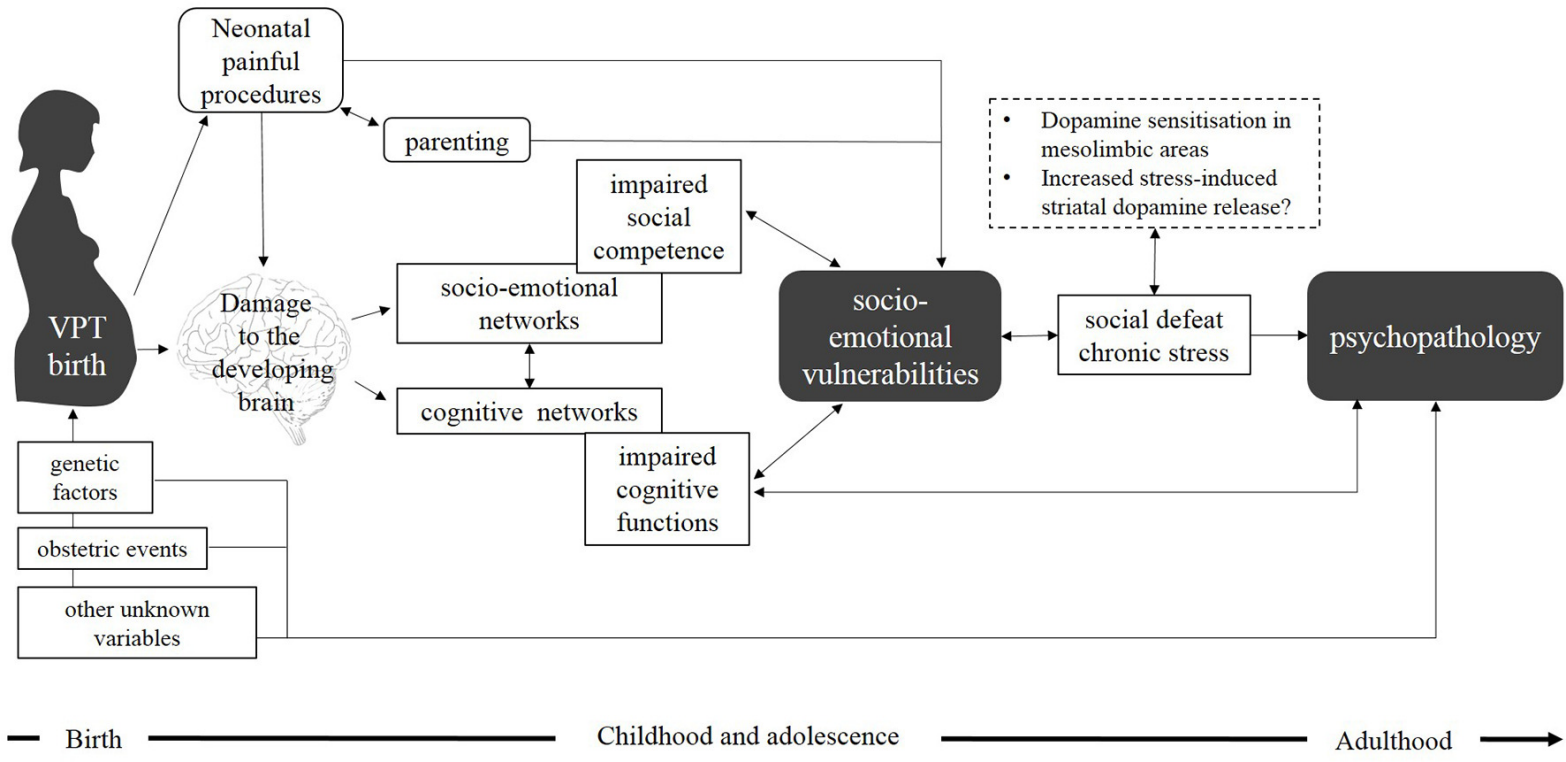
**Adapted from Healy et al. (2013) an integrative model showing biological and environmental factors underlying the association between very preterm birth, socio-emotional vulnerabilities, and psychopathology**. According to the model, VPT birth (caused by a combination of genetic factors, obstetric events, and other variables) leads to brain alterations in both socio-emotional and cognitive networks. These alterations might underlie socio-emotional vulnerabilities in childhood (possibly due to both deficits in social competence or to more general impaired cognitive functions). Painful procedures and stress experienced during the neonatal period may also impact the development of subplate neurons and preoligodendrocytes, resulting in alterations in brain microstructure. Parenting and parental mental health may mediate the effect of these early adverse events and act either as protective or exacerbating risk factors. Children and adolescents at socio-emotional risk may then be exposed to experiences of social exclusion and social victimization (social defeat and chronic social stress), which have been associated with dopamine sensitization in mesolimbic areas and increased stress-induced striatal dopamine release. We propose that dopamine dysfunction may mediate the association between socio-emotional vulnerabilities and psychopathology and contribute to increased risk of developing psychiatric morbidity in adulthood. In this model we have included a direct reciprocal link between impaired cognitive functions and psychopathology bypassing an intermediate emotional vulnerability stage.

Here we propose an integrative model that takes into account the reciprocal effect of interacting aspects of development (Figure 2). In this model, early brain alterations associated with preterm birth challenge the typical trajectory of brain development and (directly or indirectly) affect socio-emotional development. The effect of neonatal pain and stress may lead to disrupted development of subplate neurons and preoligodendrocytes, which lead to subsequent alterations in brain microstructure. These processes may occur in addition to perinatal brain injury associated with VPT birth and are thought to contribute to later socio-emotional disturbances.

During the first stages of development, parenting and parental mental health mediate offspring's neurodevelopmental outcomes, by acting either as protective or exacerbating risk factors. Parental stress and psychological well-being have in fact been described as being fundamental for a healthy development of self-regulatory abilities and socio-emotional development. Compromised parent-child interactions may confer a further developmental risk for socio-emotional problems.

The complex interplay between these factors place preterm individuals at increased risk for socio-emotional behavioral problems, and these in turn contribute to the risk of developing psychiatric disorder later in life. The association between behavioral problems and psychopathology is supported by the social defeat hypothesis of mental disorders, which suggests that psychiatric vulnerability is increased due to the effect of socio-emotional difficulties on children's experiences of social exclusion and social victimization. Social stress and isolation may affect the dopamine system through a process of dopamine sensitization or increased stress-induced dopamine release. This dopaminergic dysfunction has been associated with an increased risk for psychopathology. Whilst this is the framework we are using in our review, we also acknowledge research which has suggested a direct reciprocal link between impaired cognitive functions and psychopathology bypassing an intermediate emotional vulnerability stage (McGrath et al., 2015).

When considering the possible causal relationship between these factors, a large number of other variables need to be taken into account, as a series of morbidities commonly associated with preterm birth can contribute to the association between socio-emotional difficulties and psychopathology. For instance, parental psychiatric history has been described as a risk factor for both preterm birth and child's psychopathology, making it more difficult to disentangle the relative contribution of prematurity to psychiatric outcomes. Moreover, genetic factors may be included in this model, as specific genetic variants have been associated with an increased risk for psychopathology in conditions of biological risk (Cannon et al., 2002; Dean et al., 2010; Nosarti, 2013).

Biological risk may include early brain insults associated with VPT birth, such as hypoxia/ischemia and periventricular leukomalacia (Volpe, 2009). Animal models suggest early brain injury leads to altered prefrontal-hippocampal development leading in turn to increased striatal dopamine release (Mittal et al., 2008). In this context, neurodevelopmental alterations may produce lasting effects on dopamine function, increasing mesolimbic dopamine response to stressful stimuli (Boksa and El-Khodor, 2003; Lipska, 2004; Boksa, 2010). As previously described, dopaminergic dysfunction is associated with an increased susceptibility to environmental stressors and an increased risk of psychopathology. According to these studies, dopamine dysregulation (following perinatal brain lesions) provides a rational mechanism linking premature brain injuries to psychopathology, but further work is clearly needed to elucidate exactly how perinatal lesions can affect the dopamine system and in turn increase the risk for socio-emotional and psychiatric problems.

## Conclusions

The studies summarized in this article indicate that socio-emotional disturbances are highly prevalent among samples of individuals who were born very preterm and that preterm birth represents an independent risk factor for psychiatric disorder.

In order to increase our understanding of the association between VPT birth and vulnerability to developing socio-emotional and psychiatric problems we explored the complex interplay between biological vulnerabilities and environmental influences, including functional and structural brain alterations, neonatal pain and stress and non-optimal parenting strategies. We hypothesized that the association between socio-emotional difficulties and psychopathology may be mediated by a repeated experience of psychosocial stress and social defeat, resulting in lasting effects on dopaminergic function, leading to behavioral impairments.

A broader understanding of the complex interactions amongst biological and environmental factors remains the goal of further investigations. The elucidation of the mechanisms linking preterm birth, socio-emotional and psychiatric problems could provide an evidence-based rationale for developing and delivering new effective interventions meant to specifically reinforce protective factors (e.g., intervention on parental stress) or to target specific factors found to be precursors of socio-emotional and psychiatric difficulties in preterm born individuals.

New findings on early markers of outcome will help us explain the cascade of socio-emotional behavioral difficulties often described in preterm born individuals, will provide a better understanding of their brain substrates and will open the way to new effective contributions to support child development.

## Author contributions

AM: Study conception and design, data interpretation and write up. CN: Study conception and design, data interpretation and write up.

### Conflict of interest statement

The authors declare that the research was conducted in the absence of any commercial or financial relationships that could be construed as a potential conflict of interest.
